# Chronic eosinophilic pneumonia after trastuzumab and radiation therapy for breast cancer: A case report

**DOI:** 10.1097/MD.0000000000014017

**Published:** 2019-01-04

**Authors:** Fan Jin, Shao-ting Wang

**Affiliations:** a Department of Internal Medicine,; b Department of Respiratory Medicine, Peking Union Medical College Hospital, Chinese Academy of Medical Science and Peking Union Medical College, Beijing, China.

**Keywords:** breast cancer, chronic eosinophilic pneumonia, radiation therapy, trastuzumab

## Abstract

**Rationale:**

Chronic eosinophilic pneumonia (CEP) is rare and an idiopathic disorder. The disease has been associated with drugs, infection, or irradiation, and its relationship with asthma remains unclear.

**Patient concerns:**

We reported a case of a 49-year-old female patient after trastuzumab and radiation therapy for breast cancer. Two months after radiation treatment, the patient complained of productive cough, progressive breathlessness, occasional wheezing, and left pectoralgia.

**Diagnoses:**

Computed tomography (CT) scan revealed infiltrates in lungs. Without evidence of infection, marked increased eosinophils in a transbronchial biopsy performed from the left upper lobe confirmed the diagnosis of CEP after trastuzumab and radiation therapy.

**Interventions:**

The patient was started with oral prednisone at 0.5 mg/kg/day.

**Outcomes:**

A CT scan of the chest obtained 2 weeks after steroid treatment showed diminishment of the lesions, and at the 6-month follow-up, the patient had no complaints of discomfort with no relapse of pulmonary lesions.

**Lessons:**

Physicians should consider CEP as a diagnosis in patients who have had previous exposure to trastuzumab and radiation therapy, especially with a history of asthma. Timely diagnosis and treatment may benefit these patients.

## 1 Introduction

Chronic eosinophilic pneumonia (CEP) is an idiopathic disorder characterized by an abnormal and marked accumulation of eosinophils in the interstitium and alveolar spaces of the lung.^[1]^ CEP is a rare disorder, in registries of interstitial lung disease (ILD) in Europe, which accounts for up to 2.5% of cases of ILD.^[2]^ Clinical manifestations are nonspecific with subacute to chronic respiratory symptoms. It is usually idiopathic, however, it has been associated with drugs or toxin, parasite or fungi, irradiation for cancer, and rheumatoid arthritis.^[3–5]^ In this case, a female patient with a history of asthma and breast cancer developed CEP two months after she completed radiation and trastuzumab therapy.

## 2 Case report

**Figure 1:**
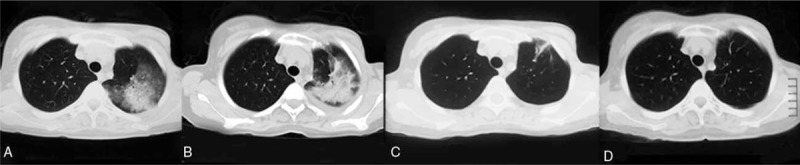
(A) CT showing a left upper lobe consolidation. (B) CT showing the consolidation increased and newly seen small-sized pleural effusion. (C) CT showing the consolidations diminished after steroid treatment for 2 weeks. (D) At 6-month follow-up, CT showing no relapse of pulmonary lesions.

A 49-year-old female with a medical history significant for left-sided breast cancer underwent lumpectomy in 2017, for which the pathology was ductal carcinoma, HER2 positive. Then, she was treated with trastuzumab (8 mg/kg loading dose, then 6 mg/kg every 3 weeks intravenously) and oral capecitabine (1000 mg/m^2^ twice a day on days 1–14 every 3 weeks) for 7 cycles from September 2017 to February 2018. During chemotherapy, a course of radiotherapy was performed, delivering 50 Gy in 25 fractions to left chest wall and supraclavicular fossa (2 Gy every fraction and 5 fractions per week) from November 17 to December 22, 2017. Two months after radiation treatment, the patient complained of productive cough and progressive breathlessness, occasional wheezing, and left pectoralgia. Her chest X-ray showed infiltrates in the left apical segment and was prescribed ipratropium inhalers and antibiotics. With no improvement in her symptoms, the computed tomography (CT) scan of the chest (Fig. 1A) revealed a left upper lobe consolidation. Half a month later, the range and density of the consolidation increased, and the left pleural effusion was newly seen (Fig. 1B).

On review of systems, the patient reported suffering from nocturnal sweats but no fevers, no change in appetite, and no weight loss. She had a full-time job as an office worker and denied any significant environmental exposure history. She is a never smoker with a 6-year past medical history of well-controlled asthma.

**Figure 2:**
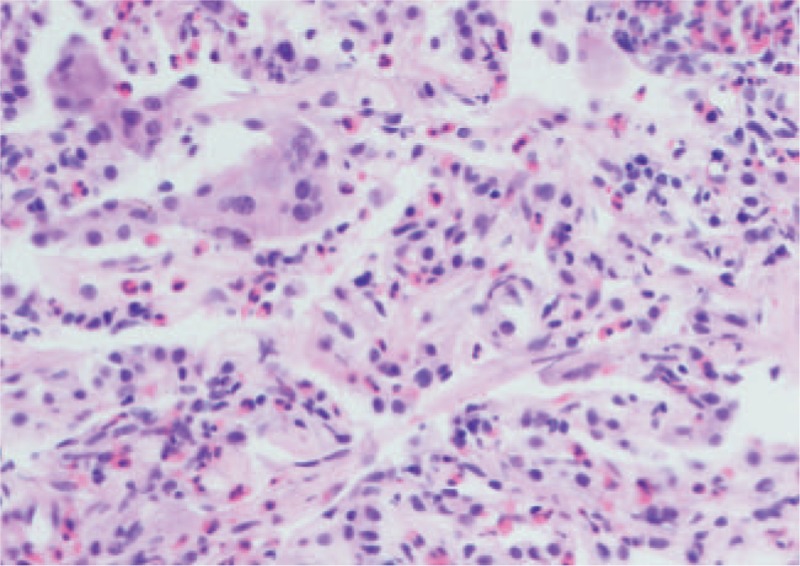
Transbronchial biopsy showing eosinophilic infiltrates within lung parenchyma. No vasculitis or microorganisms were seen; H&E, ×200 magnification.

Laboratory studies revealed 56% eosinophils (6.16 × 10^9^/L) in peripheral blood, IgE 154.0 kU/L. Her blood biochemical profiles as well as serum immunoglobulins were all unremarkable. Infectious disease etiologies workup including serologies for aspergillus, filarial worms, lungworms, cysticercosis, and trichinella spiralis was negative. Stool examinations for ova and parasites were negative. Vasculitides and connective tissue diseases workup including antinuclear antibody, anti-double-stranded DNA, rheumatoid factor, and anti-neutrophil cytoplasmic antibody was negative. Bone marrow biopsy and FIP1L1-PDGFR alpha test excluded hematological diseases such as myeloproliferative neoplasms and hypereosinophilic syndrome. Pulmonary function tests revealed forced expiratory volume in the first second of 2.88 L (97.0% predicted), FEV_1_/FVC ratio of 73.73%, total lung capacity of 4.68 L (96.9% predicted), and diffusing capacity for carbon monoxide of 6.41 mmol/min/kPa (79.8% predicted). She underwent bronchoscopy, and bronchoalveolar lavage (BAL) fluid demonstrated eosinophil count of 85%. A transbronchial biopsy performed from the left upper lobe was suggestive of eosinophilic pneumonia with marked increased eosinophils without evidence of vasculitis, malignancy, or infection (Fig. 2). The diagnosis of chronic eosinophilic pneumonia (CEP) was made based on peripheral eosinophilia, pulmonary consolidation with pleural effusion, high percentage of BAL eosinophils, the finding of eosinophilic pneumonia on transbronchial biopsy, and the absence of other causes of eosinophilia. Our patient was given oral prednisone at 0.5 mg/kg/day and all antibiotics were discontinued. She had an excellent rapid clinical improvement. A CT scan of the chest (Fig. 1C) obtained 2 weeks after steroid treatment showed diminishment of the consolidations. Her prednisone was tapered slowly over 1 month and consequently stopped. At 6-month follow-up, the patient had no complaints of discomfort with no relapse of pulmonary lesions (Fig. 1D).

## 3 Discussion

The pathophysiology of CEP remains incompletely delineated, though elevations of several cytokine, chemokine, and immunomodulatory products in studies of BAL fluid existed.^[1,3]^ Women develop CEP about twice as often as men and a majority of patients are nonsmokers. A third to a half of affected patients have a history of asthma.^[6]^ The diagnosis of CEP is typically based on the combination of clinical presentation, and typical symptoms include a productive cough, fever, breathlessness, weight loss, and night sweats, occurring over 2 months.^[7]^ Chest imaging may show predominantly peripheral or pleural-based opacities. The BAL eosinophil count is always more than 25% in CEP.^[6–8]^ CEP is usually a diagnosis of exclusion and the differential diagnosis includes acute eosinophilic pneumonia, allergic bronchopulmonary aspergillosis, infectious causes, eosinophilic granulomatosis with polyangiitis, or hypereosinophilic syndrome.^[9]^ Lung biopsy is not necessary unless the BAL does not show eosinophilia, the chest imaging features are atypical, or the patient does not respond promptly to systemic glucocorticoid therapy. Response to steroids aids in confirming the diagnosis and is usually with dramatic improvement in symptoms within 24–48 h.^[8]^

Therapy is indicated once the diagnosis of CEP has been made, as fewer than 10% of patients with CEP spontaneously recover. In addition, CEP occasionally leads to irreversible fibrosis, although death secondary to CEP is extremely unusual.^[1,7]^ Initial therapy consists of oral prednisone at a dose of 0.5 mg/kg per day. We usually continue the initial dose for 2 weeks after the complete resolution of symptoms and abnormal plain chest radiographic (usually 4–6 weeks).^[9]^ Symptomatic or radiographic recurrence is common (50–80% of cases) either after cessation of therapy, or less commonly, with tapering of the glucocorticoid dose. In managing a relapse, the prednisone dose can be restored to 0.5 mg/kg per day. Treatment usually lasts at least 3 months, usually 6–9 months. Occasionally, glucocorticoid therapy continues indefinitely.^[9]^ Alternative therapies have been explored for patients with recurrent flares of CEP that are associated with radiographic opacities, including high-dose inhaled glucocorticoids and omalizumab (monoclonal antibody to IgE).^[10,11]^ Inhaled glucocorticoids (1000–1500 μg per 24 h) have been reported to be effective in CEP. Inhaled glucocorticoids are not recommended as an initial or monotherapy, but may help in reducing the maintenance dose of oral glucocorticoid.^[10]^

Our patient met the diagnosis of CEP, and the therapeutic effects of glucocorticoid have proved the diagnosis. After exclusion of other causes, the pneumonitis appeared to be the consequence of trastuzumab or radiotherapy. Radiation therapy has been associated with the development of eosinophilic pneumonia, especially in those who have underlying allergies such as asthma.^[12,13]^ Cottin and Chaaban successively reported cases of patient with CEP in breast patients post-radiation therapy, and all patients rapidly improved with oral corticosteroids without sequelae.^[13,14]^ In addition to radiotherapy, triggers, such as drugs, toxins, and environmental exposure, are usually needed.^[13,15]^ No case of trastuzumab causing CEP has been reported, but there are reports of trastuzumab related to pulmonary interstitial disease in the NSABP B-31 and N9831 trials, which revealed that 9 patients in the trastuzumab group had interstitial pneumonitis.^[16]^ Physicians should be aware of a causative association between trastuzumab and pulmonary toxicity, and consider CEP as a diagnosis in patients who have had previous exposure to trastuzumab and radiation therapy for breast cancer.

## Acknowledgments

The authors thank Dr Dachun Zhao of Department of Pathology, PUMCH for his valuable help and advice.

## Author contributions

**Writing – original draft:** Fan Jin, Shaoting Wang.

**Writing – review & editing:** Fan Jin, Shaoting Wang.

## References

[1] Allen JN Davis WB Eosinophilic lung diseases. Am J Respir Crit Care Med 1994;150:1423–38. 7952571 10.1164/ajrccm.150.5.7952571

[2] Thomeer MJ Costabe U Rizzato G Comparison of registries of interstitial lung diseases in three European countries. Eur Respir J Suppl 2001;32:114s–8s. 11816817

[3] Akuthota P Weller PF Eosinophilic pneumonias. Clin Microbiol Rev 2012;25:649–60. 23034324 10.1128/CMR.00025-12 PMC3485750

[4] Jaimes-Hernández J Mendoza-Fuentes A Meléndez-Mercado CI Chronic eosinophilic pneumonia: autoimmune phenomenon or immunoallergic disease? Case report and literature review. Reumatol Clin 2012;8:145–8. 22196999 10.1016/j.reuma.2011.09.005

[5] Campos LE Pereira LF Pulmonary eosinophilia. J Bras Pneumol 2009;35:561–73. 19618037 10.1590/s1806-37132009000600010

[6] Sveinsson OA Isaksson HJ Gudmundsson G [Chronic eosinophilic pneumonia in Iceland: clinical features, epidemiology and review]. Laeknabladid 2007;93:111–6. 17277407

[7] Jederlinic PJ Sicilian L Gaensler EA Chronic eosinophilic pneumonia. A report of 19 cases and a review of the literature. Medicine (Baltimore) 1988;67:154–62. 3285120 10.1097/00005792-198805000-00002

[8] Järvenpää R Holli K Pitkänen M Radiological pulmonary findings after breast cancer irradiation: a prospective study. Acta Oncol 2006;45:16–22. 16464791 10.1080/02841860500334921

[9] Marchand E Reynaud-Gaubert M Lauque D Idiopathic chronic eosinophilic pneumonia. A clinical and follow-up study of 62 cases. The Groupe d’Etudes et de Recherche sur les Maladies “Orphelines” Pulmonaires (GERM“O”P). Medicine (Baltimore) 1998;77:299–312. 9772920 10.1097/00005792-199809000-00001

[10] Minakuchi M Niimi A Matsumoto H Chronic eosinophilic pneumonia: treatment with inhaled corticosteroids. Respiration 2003;70:362–6. 14512670 10.1159/000072898

[11] Cazzola M Mura M Segreti A Eosinophilic pneumonia in an asthmatic patient treated with omalizumab therapy: forme-fruste of Churg-Strauss syndrome? Allergy 2009;64:1389–90. 19392997 10.1111/j.1398-9995.2009.02061.x

[12] Guerriero G Battista C Montesano M Unusual complication after radiotherapy for breast cancer bronchiolitis obliterans organizing pneumonia case report and review of the literature. Tumori 2005;91:421–3. 16459640 10.1177/030089160509100508

[13] Cottin V Frognier R Monnot H Chronic eosinophilic pneumonia after radiation therapy for breast cancer. Eur Respir J 2004;23:9–13. 14738224 10.1183/09031936.03.00071303

[14] Chaaban S Salloum V Chronic eosinophilic pneumonia in a breast cancer patient post-radiation therapy: a case report. Respir Care 2014;59:e81–3. 24026185 10.4187/respcare.02458

[15] Cordier JF Schwarz MI King TE Eosinophilic pneumonias. Interstitial Lung Disease. 4th edn. Hamilton, Ontario, B.C.: Decker Inc; 2003 657–700.

[16] Romond EH Perez EA Bryant J Trastuzumab plus adjuvant chemotherapy for operable HER2-positive breast cancer. N Engl J Med 2005;353:1673–84. 16236738 10.1056/NEJMoa052122

